# Routine laboratory composite indices and geriatric multisyndrome burden: a head to head evaluation against serum albumin

**DOI:** 10.3389/fmed.2026.1931199

**Published:** 2026-09-09

**Authors:** Kübra Erdoğan, Rıdvan Erten

**Affiliations:** 1 Department of Internal Medicine, Division of Geriatrics, Ankara Bilkent City Hospital, Ankara, Türkiye; 2 Department of Internal Medicine, Division of Geriatrics, Faculty of Medicine, Kocaeli University, Kocaeli, Türkiye

**Keywords:** geriatric assessment, HALP score, multimorbidity, nutritional status, prognostic nutritional index, serum albumin

## Abstract

**Introduction:**

Routine blood tests are often combined into composite indices meant to summarize nutritional, inflammatory, or metabolic status in a single value. Whether these indices reflect the broad syndrome burden seen in geriatric outpatients is unclear, since most of the supporting evidence comes from oncological or surgical cohorts.

**Methods:**

We reviewed the records of 289 patients seen consecutively at a geriatric outpatient clinic over an approximately one-year period and calculated thirteen indices from the first available laboratory panel. The outcome was multisyndrome burden, scored across ten geriatric syndromes, with a burden of four or more defined as high. Each index was entered into a separate logistic regression adjusted for age, sex, comorbidity, and polypharmacy, and the thirteen *p*-values were corrected for multiple testing with the Benjamini–Hochberg procedure.

**Results:**

Only the Prognostic Nutritional Index (PNI) and the HALP score remained associated with high burden after correction (adjusted odds ratio per standard deviation 0.55 and 0.64, respectively); none of the inflammatory or metabolic indices reached significance in the full cohort, although the neutrophil-to-lymphocyte ratio and the systemic immune-inflammation index did so in the subgroup in whom all ten components were assessed. Removing the two activity-of-daily-living measures from the outcome left neither association significant, which places the relationship mainly on functional status rather than on an independent inflammatory pathway. Neither index discriminated better than serum albumin measured alone (PNI, area under the curve 0.679 vs. 0.677, DeLong *p* = 0.90; HALP, 0.654 vs. 0.677, *p* = 0.52).

**Discussion:**

Taken together, the results argue against treating composite laboratory indices as independent markers of geriatric vulnerability: their apparent value largely reflects the albumin and functional components already captured in routine assessment. A low PNI or HALP may still draw attention to a patient who warrants closer functional evaluation, but should not replace direct geriatric assessment.

## Introduction

People are living longer, and a growing share of the patients seen in everyday clinical practice are of advanced age (1). Advanced age is associated with a cluster of problems that do not map onto a single organ system (falls, incontinence, cognitive decline, malnutrition, depression), and these frequently co-occur. Because they share risk factors and feed back on one another, a patient seldom has only one (2, 3). Disability and adverse outcomes track the number of concurrent syndromes more closely than any single diagnosis (4).

A simple early marker of such vulnerability would be clinically useful. Routine laboratory values are appealing for the task: they are inexpensive, quantitative, and already on file, and some of them flag higher mortality in older adults even when they sit within the reference range (5). Over the last decade attention has moved from single analytes to composite indices that fold several routine values into one number. The Prognostic Nutritional Index (PNI), derived from serum albumin and lymphocyte count, began in surgical patients and was later recast as a combined nutritional and immune measure (6, 7). The HALP score, which combines hemoglobin, albumin, lymphocyte, and platelet counts, came out of gastric cancer work and has since been used in oncological and cardiovascular cohorts (8, 9). Related inflammation-based ratios and metabolic indices have also been proposed.

Almost all of this evidence comes from oncology, surgery, or intensive care. Whether the indices carry useful information about the diffuse syndrome burden of a general geriatric outpatient, a person who is not acutely ill but has progressive functional and nutritional deficits, has had far less scrutiny, and next to none in a Turkish outpatient setting. This gap has direct clinical consequences: a clinician facing such a patient needs to know whether a low index value adds anything to what a basic panel and a brief functional assessment already reveal.

We therefore examined how thirteen routine laboratory indices relate to multisyndrome burden in older adults attending a geriatric outpatient clinic. Two further questions followed: which syndrome components carried any association we found, and whether the better-performing indices discriminated beyond serum albumin measured on its own. We did not set out to build a screening tool. The question was narrower: whether these indices behave as independent markers of burden, or simply restate information the clinician already holds.

## Materials and methods

### Study design and participants

This was a single-center, retrospective, cross-sectional review of consecutive patients aged 65 years or older seen at the Geriatrics Outpatient Clinic of Ankara Bilkent City Hospital, each of whom underwent comprehensive geriatric assessment with concurrent laboratory testing at the same visit. Records were examined retrospectively over an approximately one-year period, with first visits spanning June 2025 to April 2026, ending before ethics approval was obtained on 13 May 2026. The laboratory panel analyzed was the first set of results recorded at that visit, drawn on the same day as the assessment; no interval was permitted between sampling and assessment. Of 295 patients initially identified, 6 were excluded because more than half of the laboratory panel was missing, leaving 289 for analysis. The study is reported in line with the STROBE statement for observational research (10); participant flow is shown in Supplementary Figure S1.

### Data sources and variables

Age, sex, comorbidities, medication counts, the first laboratory values recorded at the visit, and the results of comprehensive geriatric assessment were retrieved from the hospital's electronic and printed records. Comorbidity burden was summarized with the Charlson Comorbidity Index (11). Polypharmacy was defined as the regular use of five or more medications. Laboratory values were used as measured; for index calculation, hemoglobin was expressed in g/L, and albumin in g/L for HALP and in g/dL for PNI and the C-reactive protein–albumin ratio. C-reactive protein was recorded by the laboratory in mg/dL and multiplied by ten to give mg/L for the two indices derived from it. C-reactive protein values recorded as zero were replaced by the smallest observed positive value when the CALLY index was computed.

### Outcome definition

The outcome was multisyndrome burden, scored across ten geriatric syndromes: falls, delirium, urinary incontinence, osteoporosis, sleep problems, dependency in activities of daily living (ADL), dependency in instrumental activities of daily living (IADL), cognitive impairment, risk of malnutrition, and depression. Each component was coded present or absent against a prespecified definition, and the composite was their sum, ranging from 0 to 10. Components that were not assessed at the index visit were counted as absent; because ascertainment was incomplete for the cognitive and mood instruments in particular, a sensitivity analysis restricted to patients in whom all ten components were recorded is reported in the Supplementary Material. Functional, cognitive, nutritional, and mood status were assessed with the Katz ADL index, the Lawton–Brody IADL scale, the standardized Mini-Mental State Examination, the Mini Nutritional Assessment–Short Form, and the 15-item Geriatric Depression Scale, using validated Turkish versions of these instruments (12–20). High burden was defined as four or more syndromes. This cut-off falls near the cohort median and identifies a clinically higher-burden phenotype; alternative thresholds were examined in sensitivity analyses. The cognitive component denotes screen-level cognitive impairment, defined by a standardized Mini-Mental State Examination score below 24; it does not represent a dementia diagnosis. Dementia was recorded separately as a pre-existing clinical diagnosis made according to DSM-5 criteria, retrieved from the medical records, and was analyzed as a comorbidity rather than as an outcome component; it was not derived from the Mini-Mental State Examination. Full operational definitions of each component are given in Supplementary Table S1.

### Composite laboratory indices

Thirteen indices were computed from the index laboratory panel. Four were nutrition- or inflammation-based: PNI, HALP, the C-reactive protein–albumin–lymphocyte (CALLY) index, and the C-reactive protein-to-albumin ratio (CAR). Five were classical inflammation-based: the neutrophil-to-lymphocyte ratio (NLR), systemic immune-inflammation index (SII), systemic inflammatory response index (SIRI), pan-immune-inflammation value (PIV), and monocyte-to-HDL ratio (MHR). Four were metabolic or atherogenic: the triglyceride-glucose index (TyG), atherogenic index of plasma (AIP), cholesterol–HDL–glucose index (CHG), and non-HDL/HDL ratio (NHHR). Each index was converted to a *z*-score before modeling, so that effect sizes are expressed per standard deviation and are comparable across indices. Formulas and unit conventions are listed in Supplementary Table S2.

### Statistical analysis

Continuous variables are summarized as median and interquartile range, since only one of them was normally distributed on the Shapiro–Wilk test (21); groups were compared with the Mann–Whitney *U* test, except for the single normally distributed variable, for which the Welch *t*-test was used, and categorical variables, compared with the chi-square or Fisher exact test. Because several components of the outcome are themselves scale scores, *p*-values for those variables were not reported in the descriptive table, to avoid a circular comparison.

The primary analysis modeled high burden (four or more syndromes vs. fewer) on each index in turn, using binary logistic regression adjusted for age, sex, Charlson Comorbidity Index, and polypharmacy. These four covariates were fixed on clinical grounds; stepwise selection was not used (22). With five parameters and 125 patients in the smaller outcome group of the complete-case model (*n* = 278), there were roughly 25 events per variable, comfortably above the usual minimum for stable estimation (23, 24). The thirteen indices were selected *a priori* before outcome modeling on the basis of their previous use as routine laboratory-derived nutritional, inflammatory, or metabolic composite markers. No index was selected on the basis of its observed association with the outcome. Accordingly, this was interpreted as a comparative, hypothesis-screening evaluation across established indices rather than as confirmatory validation of a single biomarker; the thirteen index *p*-values were corrected with the Benjamini–Hochberg false discovery rate procedure to control multiplicity, with a corrected *q*-value below 0.05 taken as significant (25). Further sensitivity analyses, specified after the primary analysis and reported as *post hoc*, examined whether the findings depended on the composition of the outcome or on residual confounding. Individual components were removed from the composite. Dementia was added as a further covariate and, separately, patients with dementia were excluded. Patients with active malignancy and those with aspartate or alanine aminotransferase above 40 U/L were excluded. Statin use was added to the covariate set for the lipid-derived indices, polypharmacy was entered as an eleventh outcome component, and the comorbidity score was reconstructed under alternative definitions. Two further descriptive analyses examined the relationship between comorbidity and syndrome burden: the Spearman rank correlation between the Charlson index and the burden count, and a logistic model of high burden on the Charlson index adjusted for age, sex, and polypharmacy. Collinearity was checked with the variance inflation factor; the highest value, 3.37, was below the conventional threshold.

For the indices that survived correction, we compared their discrimination with that of serum albumin alone using the areas under the receiver operating characteristic curves and the DeLong test (26). To locate the source of any association, we refitted the primary models after dropping selected components from the outcome — most importantly the two functional measures, ADL and IADL — and after varying the high-burden threshold. Secondary analyses treating the syndrome count as continuous (Poisson) and modeling prevalence ratios (modified Poisson) are reported in the Supplementary Material. Analyses were run in Python (statsmodels and scikit-learn), and *p*-values are two-sided.

## Results

### Cohort characteristics

The 289 patients had a median age of 78 years, and 173 (59.9%) were women. High multisyndrome burden was present in 157 (54.3%). Patients with high burden were older, carried more comorbidity, and were more often on five or more medications; dementia was nearly six times as common among them as in the low-burden group. Of the fourteen comorbidities recorded, only dementia and Parkinson disease differed significantly between the burden groups; hypertension, diabetes, coronary artery disease, chronic obstructive pulmonary disease, heart failure, chronic kidney disease, and malignancy did not. Their laboratory profile was also poorer, with lower hemoglobin, lymphocyte count, and albumin, and correspondingly lower PNI and HALP. C-reactive protein was marginally higher in the high-burden group (*p* = 0.039), but the distribution was compressed against the reporting floor in both groups and the difference has little quantitative meaning. Characteristics by burden group are shown in Table 1.

**Table 1:** Characteristics of the cohort by multisyndrome burden (*n* = 289).

Variable	Total (*n* = 289)	Low burden <4 (*n* = 132)	High burden ≥4 (*n* = 157)	p
Age, years, median (IQR)	78 (72–83)	75 (71–81)	79 (74–84)	<0.001
Female, *n* (%)	173 (59.9)	71 (53.8)	102 (65.0)	0.070
Charlson index, median (IQR)	1 (0–2)	1 (0–2)	1 (1–2)	0.010
Diabetes mellitus, *n* (%)	106 (36.7)	45 (34.1)	61 (38.9)	0.475
Dementia, *n* (%)	50 (17.3)	6 (4.5)	44 (28.0)	<0.001
Hypertension, *n* (%)	199 (68.9)	91 (68.9)	108 (68.8)	1.000
Coronary artery disease, *n* (%)	72 (24.9)	34 (25.8)	38 (24.2)	0.867
Hypothyroidism, *n* (%)	43 (14.9)	16 (12.1)	27 (17.2)	0.287
COPD, *n* (%)	33 (11.4)	15 (11.4)	18 (11.5)	1.000
Benign prostatic hyperplasia, *n* (%)	30 (10.4)	12 (9.1)	18 (11.5)	0.642
Cerebrovascular disease, *n* (%)	21 (7.3)	7 (5.3)	14 (8.9)	0.350
Depression (clinical diagnosis), *n* (%)	21 (7.3)	5 (3.8)	16 (10.2)	0.063
Congestive heart failure, *n* (%)	16 (5.6)	8 (6.1)	8 (5.1)	0.931
Parkinson disease, *n* (%)	14 (4.9)	1 (0.8)	13 (8.3)	0.002
Malignancy, *n* (%)	14 (4.9)	6 (4.5)	8 (5.1)	1.000
Chronic kidney disease, *n* (%)	13 (4.5)	5 (3.8)	8 (5.1)	0.803
Hyperthyroidism, *n* (%)	3 (1.0)	1 (0.8)	2 (1.3)	1.000
Polypharmacy (≥5 drugs), *n* (%)	165 (57.1)	62 (47.0)	103 (65.6)	0.002
Hemoglobin, g/dL, median (IQR)	13.0 (11.8–14.1)	13.3 (12.4–14.4)	12.6 (11.4–13.8)	<0.001
Lymphocyte count, 10^9^/L, median (IQR)	1.69 (1.32–2.20)	1.87 (1.42–2.27)	1.56 (1.20–2.01)	<0.001
C-reactive protein, mg/dL, median (IQR)	0.005 (0.000–0.020)	0.001 (0.000–0.010)	0.010 (0.000–0.020)	0.039
Albumin, g/L, median (IQR)	43.0 (40.8–45.0)	44.0 (42.0–45.5)	42.0 (40.0–44.0)	<0.001
PNI, mea*n* ± SD	51.3 ± 6.5	53.4 ± 6.3	49.6 ± 6.1	<0.001
HALP, median (IQR)	36.6 (25.0–50.1)	43.0 (29.9–54.9)	30.6 (22.0–45.2)	<0.001

Data are *n* (%) or median (IQR). Between-group comparisons used the Mann–Whitney *U* test (continuous variables), the Welch *t*-test (PNI), and the chi-square or Fisher exact test (categorical variables).

IQR, interquartile range; PNI, Prognostic Nutritional Index; HALP, hemoglobin–albumin–lymphocyte–platelet score.

### Association of the indices with high burden

**Figure 1:**
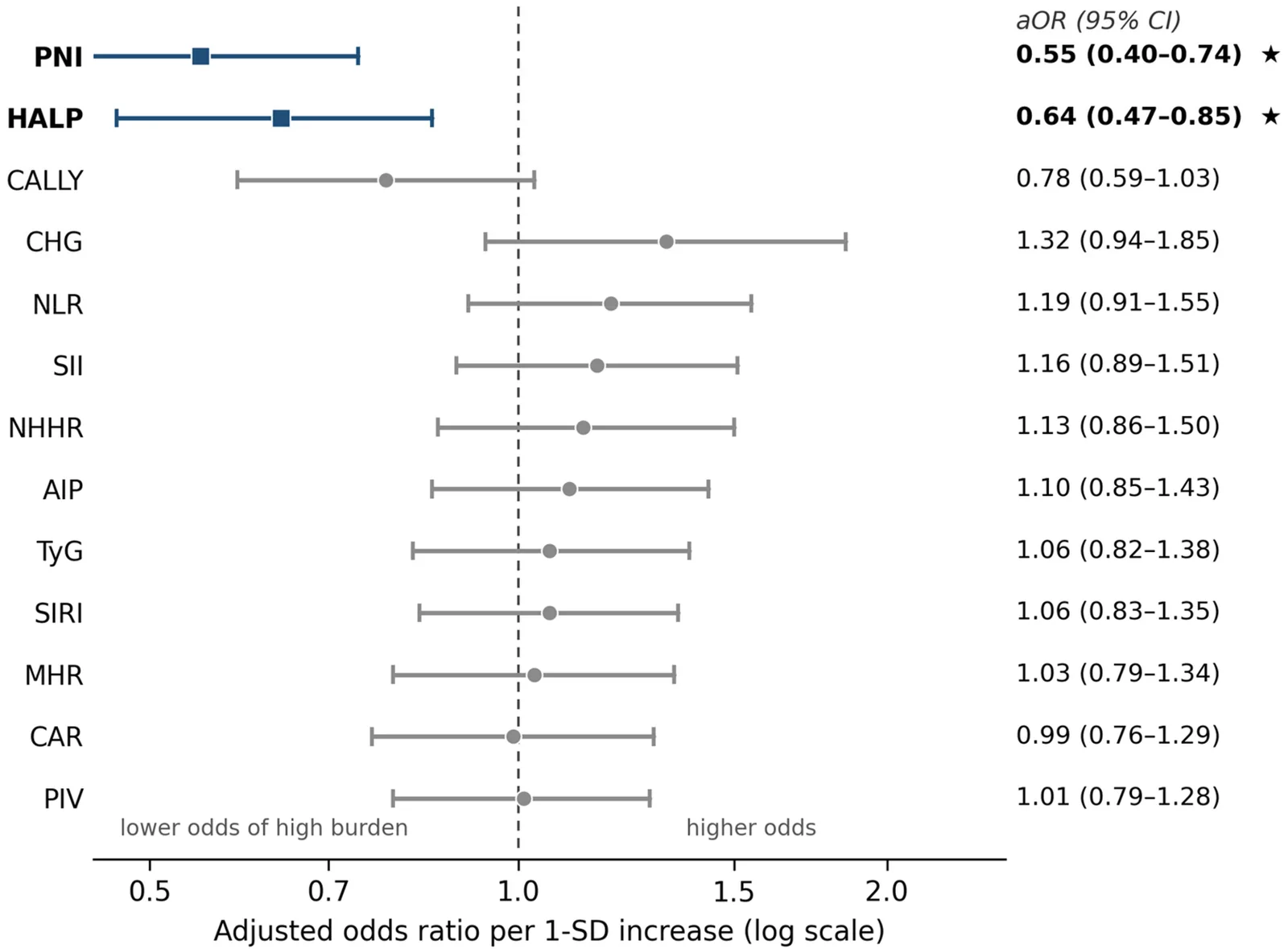
Adjusted odds ratios per one-standard-deviation increase for the thirteen laboratory indices, ordered by *p*-value. Each model was adjusted for age, sex, Charlson Comorbidity Index, and polypharmacy. Blue squares indicate PNI and HALP, which remained significant after Benjamini–Hochberg correction; grey circles indicate non-significant indices. Significant indices are also marked with a star. The dashed vertical line marks an odds ratio of 1.

After adjustment and false discovery rate correction, two of the thirteen indices were associated with high burden. Each standard-deviation increase in PNI carried roughly half the odds of high burden (adjusted odds ratio 0.55, 95% CI 0.40–0.74, q = 0.001), and each standard-deviation increase in HALP about two-thirds the odds (adjusted odds ratio 0.64, 95% CI 0.47–0.85, q = 0.016). The other eleven indices, the classical inflammatory ratios and the metabolic indices alike, did not reach significance after correction; in the full cohort none crossed even the nominal 0.05 threshold, and their adjusted odds ratios clustered around one. The full set of estimates is given in Table 2 and shown in Figure 1.

**Table 2:** Adjusted associations of the thirteen indices with high multisyndrome burden (binary logistic regression).

Index	*n*	aOR per SD	95% CI	*p*	FDR-q
PNI	278	0.55	0.40–0.74	<0.001	0.001
HALP	278	0.64	0.47–0.85	0.003	0.016
CALLY	250	0.78	0.59–1.03	0.080	0.349
CHG	261	1.32	0.94–1.85	0.112	0.365
NLR	288	1.19	0.91–1.55	0.204	0.530
SII	288	1.16	0.89–1.51	0.276	0.598
NHHR	264	1.13	0.86–1.50	0.373	0.692
AIP	264	1.10	0.85–1.43	0.470	0.764
TyG	270	1.06	0.82–1.38	0.655	0.856
SIRI	288	1.06	0.83–1.35	0.659	0.856
MHR	269	1.03	0.79–1.34	0.847	0.962
CAR	251	0.99	0.76–1.29	0.920	0.962
PIV	288	1.01	0.79–1.28	0.962	0.962

All models were adjusted for age, sex, Charlson Comorbidity Index, and polypharmacy, with the index entered as a *z*-score (per 1-SD). FDR-q, Benjamini–Hochberg false discovery rate *q*-value across the thirteen indices. The analytic sample varies by index according to data availability.

aOR, adjusted odds ratio.

### Discrimination against albumin and the source of the association

Because both surviving indices are built partly from albumin, we asked whether they added anything to albumin on its own. Both PNI and serum albumin showed only modest absolute discrimination for high multisyndrome burden (area under the curve 0.679 and 0.677, respectively), indicating limited value for case identification irrespective of the comparison between them. PNI did not discriminate better than albumin alone (DeLong *p* = 0.90), and neither did HALP, whose area under the curve was lower at 0.654 (DeLong *p* = 0.52). Formal comparison therefore gave no evidence that either composite index outperforms albumin measured on its own.

The component-removal analysis showed where the association sat. When ADL and IADL were taken out of the outcome and the score rebuilt without them, neither PNI nor HALP remained associated with high burden (Table 3). Removing the nutritional component alone left PNI significant but HALP no longer so, whereas removing both functional measures left neither index significant. The association between these indices and syndrome burden, in other words, was carried mainly by the functional-dependency items, with the nutritional component adding to it, not by any independent inflammatory or metabolic effect. The pattern held across alternative burden thresholds (Supplementary Table S3). In the subgroup in whom all ten components were recorded (*n* = 173), the PNI and HALP associations were stronger than in the full cohort (adjusted odds ratio 0.48 and 0.52), but NLR and SII also reached the corrected threshold in that subgroup (1.96 and 2.04), which they did not do in the full cohort (Supplementary Table S4).

**Table 3:** Effect of removing functional components from the outcome on the PNI and HALP associations (*post hoc*).

Outcome definition	PNI aOR (95% CI)	FDR-q	HALP aOR (95% CI)	FDR-q
Primary (10 components, ≥4)	0.55 (0.40–0.74)	0.001	0.64 (0.47–0.85)	0.016
Malnutrition item removed (9 components, ≥4)	0.64 (0.48–0.85)	0.034	0.71 (0.53–0.96)	0.163
ADL + IADL removed (8 components, ≥3)	0.78 (0.60–1.02)	0.874	0.84 (0.64–1.08)	0.874
All three removed (7 components, ≥3)	0.68 (0.51–0.91)	0.107	0.76 (0.57–1.02)	0.276

aOR, adjusted odds ratio per standard deviation; models adjusted as in Table 2. Each outcome definition was analyzed separately, and FDR-*q* values were computed across the thirteen indices within that definition. Because removing components alters both the composition of the score and the number of patients meeting the high-burden threshold, estimates are not directly comparable across rows: the seven-component definition uses a lower threshold and a different case mix from the eight-component definition, which is why its estimate is not intermediate between the others. The loss of significance when ADL and IADL are removed indicates that the association rests substantially on the functional-dependency items.

### Sensitivity analyses

Several further analyses were run to test whether the findings depended on the composition of the outcome or on measurable confounding (Table 4). Removing the cognitive item from the composite, which reduced the number of patients classified as high burden from 157 to 147 of the 289 analyzed, left both associations intact (models fitted on the 278 complete cases). Adding dementia as a further covariate, and separately excluding all patients with a diagnosis of dementia, strengthened rather than attenuated both estimates. Excluding patients with active malignancy, and excluding those with aspartate or alanine aminotransferase above 40 U/L, also left the associations in place. Entering polypharmacy as an eleventh outcome component, with the covariate set reduced accordingly, preserved both associations. For the lipid-derived indices, adding statin use to the covariate set left every estimate null (non-HDL/HDL ratio 1.14, 95% CI 0.86–1.50; CHG 1.13, 0.86–1.49; AIP 1.10, 0.84–1.43; TyG 1.05, 0.81–1.37). Because the outcome was common, prevalence ratios were also estimated directly with modified Poisson regression, and the syndrome count was modeled as a continuous outcome with Poisson regression; both gave the same pattern as the primary analysis (Supplementary Tables S5, S6).

**Table 4:** Sensitivity analyses for the PNI and HALP associations with high multisyndrome burden.

Analysis	n	PNI aOR (95% CI)	p	HALP aOR (95% CI)	p
Primary model	278	0.55 (0.40–0.74)	<0.001	0.64 (0.47–0.85)	0.003
Cognitive item removed from outcome	278	0.57 (0.43–0.77)	<0.001	0.64 (0.48–0.86)	0.003
Dementia added as covariate	278	0.53 (0.38–0.73)	<0.001	0.60 (0.44–0.83)	0.002
Patients with dementia excluded	229	0.48 (0.34–0.67)	<0.001	0.60 (0.42–0.84)	0.003
Active malignancy excluded	264	0.59 (0.43–0.80)	0.001	0.69 (0.52–0.90)	0.007
AST or ALT >40 U/L excluded	258	0.51 (0.37–0.69)	<0.001	0.59 (0.43–0.81)	0.001
Polypharmacy as eleventh component	278	0.69 (0.51–0.93)	0.016	0.65 (0.49–0.87)	0.004

All analyses in this table are *post hoc*.

Unless stated otherwise, models were adjusted for age, sex, Charlson Comorbidity Index, and polypharmacy, with the index entered as a *z*-score (per 1-SD). In the analysis entering polypharmacy as an outcome component it was removed from the covariate set.

aOR, adjusted odds ratio; AST, aspartate aminotransferase; ALT, alanine aminotransferase; CI, confidence interval; HALP, hemoglobin–albumin–lymphocyte–platelet score; PNI, Prognostic Nutritional Index.

Because the comorbidity score is a key covariate, the primary models were also refitted under five constructions of that score: the weighted index used here, an age-adjusted version, a version excluding coronary artery disease, an unweighted count of the same conditions, and a count of all fourteen recorded comorbidities. The adjusted odds ratio for PNI ranged from 0.55 to 0.56 and that for HALP from 0.63 to 0.64 across these specifications (Supplementary Table S7).

## Discussion

In a general geriatric outpatient cohort, only two of thirteen routine laboratory indices — PNI and HALP tracked multisyndrome burden once we adjusted for age, sex, comorbidity, and polypharmacy and corrected for multiple testing. None of the inflammatory or metabolic indices showed a significant association after correction. The two that did associate are both anchored in serum albumin, neither showed a clear discriminatory advantage over albumin alone, and once the functional items were removed from the outcome both associations were no longer significant. The most plausible interpretation is that these indices do not capture a separate biological axis of geriatric vulnerability; they largely restate the albumin and functional information that a routine panel and a short assessment already provide.

Composite indices are usually presented as adding value precisely by combining signals, which is what makes this result awkward for them. For broad syndrome burden in this outpatient setting, combining the components into an index added little beyond the individual parameters. The result fits what is known about albumin, which falls with both malnutrition and chronic inflammation and has long tracked functional decline in older people (27). It also fits how PNI and HALP behave elsewhere: where they are prognostic in older patients, it is usually in acute settings with a hard endpoint, such as postoperative or intensive-care cohorts, in which the nutritional signal is sharper than in a stable outpatient sample (9, 28).

The null result for the inflammatory and metabolic indices is worth examining before it is set aside. Chronic low-grade inflammation is a genuine feature of aging and contributes to frailty (29). That our inflammatory ratios did not track syndrome burden probably reflects the population studied more than the underlying biology. These were outpatients without acute illness, and C-reactive protein sat at or near the reporting floor in most of them. The metabolic indices face a different problem at this age: associations between lipids, glucose, and adverse outcomes that hold in midlife often flatten or reverse in the oldest old, where low cholesterol can mark frailty rather than health (30). Some work has linked the triglyceride-glucose index to frailty, but the size of that association shifts with the age and comorbidity profile of the sample (31). Applying midlife lipid–outcome assumptions to the oldest old is not supported by these data.

The clinical implication is correspondingly limited. A low PNI or HALP in an older outpatient may prompt formal functional and nutritional assessment but should not replace it, and it does not warrant use as a separate screening number in addition to albumin. A clinician already measuring albumin and performing a Katz or Lawton assessment gains little by also computing these composites.

Components that were not assessed were counted as absent, and ascertainment was least complete for the cognitive and mood instruments, which were missing in roughly three of every ten patients. When the analysis was restricted to the 173 patients in whom all ten components were recorded, PNI and HALP strengthened, and the neutrophil-to-lymphocyte ratio and the systemic immune-inflammation index also crossed the corrected threshold. Missingness was not random: patients without a recorded Mini-Mental State Examination were substantially more dependent in daily activities than those with one. Two mechanisms follow from this and cannot be separated with these data. Counting unassessed components as absent understates burden in the most impaired patients and would tend to attenuate real associations, so the full-cohort estimates may understate the inflammatory signal. At the same time, the fully assessed subgroup is a less impaired population, so its estimates need not generalize to the whole clinic. Either way, the null finding for the inflammatory ratios should be read as applying to the full outpatient cohort as assessed here rather than as a general absence of association, and this question would be better settled prospectively with complete ascertainment.

Comorbidity burden and syndrome burden behaved as related but distinct constructs. The Charlson index correlated only weakly with the syndrome count (Spearman rho 0.12, *p* = 0.049) and was not independently associated with high burden after adjustment for age, sex, and polypharmacy (odds ratio 1.20, *p* = 0.17). Of the fourteen comorbidities recorded, only dementia and Parkinson disease were more frequent in the high-burden group, while hypertension, diabetes, coronary artery disease, chronic obstructive pulmonary disease, heart failure, chronic kidney disease, and malignancy were not. This is consistent with the way the two constructs are built: the Charlson index weights diseases by their contribution to mortality, whereas syndrome burden reflects accumulated functional, nutritional, and cognitive deficits, and the two conditions that did track syndrome burden are precisely those with direct functional and cognitive consequences. Geriatric syndromes are recognized as a class of conditions that do not map onto single disease categories (2), the number of concurrent syndromes tracks incident disability in large cohorts independently of chronic disease burden (32), and comorbidity, frailty, and disability remain separate entities that should not be treated interchangeably (33).

Where PNI and HALP have been examined in older populations outside oncology and surgery, the reported effects are consistently modest. In a two-cohort analysis combining 5,957 participants of the West China Health and Aging Trend study with 101,655 participants of the UK Biobank, each standard-deviation increase in HALP was associated with roughly a 9% lower odds of coexisting low grip strength and cognitive impairment, and time-dependent areas under the curve for HALP-only models ranged from 0.54 to 0.58; the authors concluded that the score is best read as complementary information and not as an independent predictive tool (34). Our estimates point in the same direction, although they are larger: we observed an adjusted odds ratio of 0.64 per standard deviation for HALP against an area under the curve of 0.654, compared with roughly 0.91 and 0.54 to 0.58 in that study. The difference is what one would expect from a clinic population selected for geriatric assessment rather than a community or biobank sample. What the two studies share is the conclusion that discrimination remains modest in absolute terms. The albumin literature points the same way: hypoalbuminemia in older people is a reliable prognostic signal but a markedly non-specific one, shaped by inflammation, hydration, and hepatic and renal function as much as by nutrition (35), and albumin alone has long tracked functional decline (27). These strands converge on a single reading: the information PNI and HALP carry in a stable outpatient population is largely albumin's own, and our contribution is to test that directly: the thirteen indices were compared with one another against a single outcome in the same patients, and the two that survived correction were then benchmarked against albumin measured alone.

### Strengths and limitations

The principal strength is the simultaneous, internally consistent evaluation of thirteen indices against the same outcome in the same patients, with multiple-testing correction built in, so the comparison is not selective. Consecutive sampling over the review period limited selection bias. Several limitations bound the conclusions. First, the multisyndrome burden score was a pragmatic count of ten clinically actionable geriatric syndromes and not a validated frailty index, so the findings should not be read as evidence for frailty prediction. Second, the association between PNI or HALP and multisyndrome burden is partly structural, because the outcome included nutritional and functional components while both indices are anchored in albumin and related hematological parameters; we addressed this through component-removal analyses, but residual construct overlap is unavoidable. Third, cognitive impairment was screened with a single standardized Mini-Mental State Examination cut-off (<24) applied uniformly, without education-stratified norms or adjustment for educational attainment. Because limited formal schooling lowers Mini-Mental State Examination performance independently of cognitive status, impairment may have been overestimated in participants with little or no formal education. Fourth, the components were coded as present or absent without severity grading, which was not recorded in the source records. Fifth, ascertainment of the components was incomplete and not random. The cognitive and mood instruments were missing most often, and patients in whom the Mini-Mental State Examination was not recorded were more functionally impaired than those in whom it was: median Katz score 2 vs. 6 and median Lawton score 1 vs. 7 (both *p* < 0.001), with dementia in 32.6% vs. 10.5%. Counting unassessed components as absent will therefore have understated burden preferentially in the most impaired patients. The design is also cross-sectional and single-center, so our estimates describe association, not prognosis, and the results need confirmation in other Turkish centers. The records contain no coded diagnosis of chronic liver disease and no marker of intercurrent infection, so raised transaminases served only as a proxy and residual confounding of the albumin- and C-reactive protein-based indices cannot be excluded. C-reactive protein was at or below the reporting floor in most patients, with 32% of measured values recorded as zero, which limits inference on the two indices derived from it. The CALLY index in particular depends on how those zero values are handled: substituting the smallest observed positive value gives an adjusted odds ratio of 0.78, whereas substituting a conventional half-detection-limit value moves the estimate to unity. Neither specification reaches the corrected threshold, but the index should not be interpreted quantitatively in this dataset. and HDL and differential counts were available only in subsets, constraining the sample for some indices. Finally, the dataset carries no mortality endpoint, so we cannot speak to the prognostic value these indices may hold over time.

## Conclusion

Among thirteen routine laboratory indices, only PNI and HALP remained associated with multisyndrome burden after correction for multiple testing. Neither association remained significant once activity-of-daily-living measures were removed from the outcome, which places the association on the link between nutrition and function rather than on a separate inflammatory or metabolic pathway. In practice, a low PNI or HALP may flag a functionally vulnerable older outpatient, but neither discriminated better than serum albumin alone on formal comparison, and both are better understood as markers of existing burden than as standalone screening tests.

## Data Availability

The datasets generated and analyzed for this study are available from the corresponding author on reasonable request. They are not publicly available because of privacy and ethical restrictions.
