# Supplementary material for: Routine laboratory composite indices and geriatric multisyndrome burden: a head to head evaluation against serum albumin
Source: Front Med (Lausanne). 2026 Sep 9;13:1931199. doi: 10.3389/fmed.2026.1931199 (PMC13597418; doi:10.3389/fmed.2026.1931199)
Supplement: Supplementary file 1 [file Datasheet1.docx]

# Supplementary Material

**Routine laboratory-based composite indices and multisyndrome burden in geriatric outpatients: a cross-sectional study of thirteen indices**

Kübra Erdoğan and Rıdvan Erten

All estimates below are derived from the same complete-case models described in the main text and are adjusted, unless stated otherwise, for age, sex, Charlson Comorbidity Index, and polypharmacy, with each index entered as a z-score (per 1-SD). ★ marks a Benjamini–Hochberg false discovery rate q-value below 0.05.

## Table S1. Operational definitions of the ten syndrome components

| Component | Operational definition | Assessed, n | Not assessed, n | Present, n (%) |
| --- | --- | --- | --- | --- |
| Falls | At least one documented fall within the previous 12 months. | 287 | 2 | 83 (28.7) |
| Delirium | Active delirium at presentation or a physician-recorded delirium diagnosis. | 289 | 0 | 11 (3.8) |
| Urinary incontinence | Present on patient report and/or physician clinical assessment, or a recorded diagnosis. | 287 | 2 | 150 (51.9) |
| Osteoporosis | DXA T-score ≤ −2.5, a prior physician diagnosis, or current anti-osteoporotic treatment (any of these). | 276 | 13 | 86 (29.8) |
| Sleep problems | Present on patient report and/or physician clinical assessment, or a recorded diagnosis. | 287 | 2 | 152 (52.6) |
| ADL dependency | Katz Index < 6. | 274 | 15 | 164 (56.7) |
| IADL dependency | Lawton–Brody scale < 8. | 271 | 18 | 167 (57.8) |
| Cognitive impairment | Standardized Mini-Mental State Examination < 24. | 200 | 89 | 54 (18.7) |
| Malnutrition risk | Mini Nutritional Assessment–Short Form ≤ 11. | 264 | 25 | 139 (48.1) |
| Depression | 15-item Geriatric Depression Scale ≥ 6. | 203 | 86 | 75 (26.0) |

*High multisyndrome burden was defined as four or more components present. The Geriatric Depression Scale threshold was set at ≥ 6 rather than the ≥ 5 screening cut-off, favouring specificity within a multicomponent composite; a sensitivity analysis using ≥ 5 gave materially similar results.*

*Percentages are of the analytic sample (n = 289). Components that were not assessed were counted as absent when the composite was formed; Table S4 restricts the analysis to patients in whom all ten components were recorded. ADL, activities of daily living; DXA, dual-energy X-ray absorptiometry; IADL, instrumental activities of daily living.*

## Table S2. Formulas and unit conventions for the thirteen indices

| Abbreviation | Full name | Formula | Unit / note |
| --- | --- | --- | --- |
| PNI | Prognostic Nutritional Index | Albumin (g/L) + 5 × lymphocyte (10⁹/L) | Dimensionless; albumin and lymphocyte combined in one equation |
| HALP | Hemoglobin–albumin–lymphocyte–platelet | (Hb [g/L] × Alb [g/L] × Lymph [10⁹/L]) / platelet [10⁹/L] | Dimensionless; Hb converted from g/dL to g/L (× 10) |
| CALLY | C-reactive protein–albumin–lymphocyte index | (Alb [g/dL] × Lymph [10⁹/L]) / (CRP [mg/L] × 10) | C-reactive protein was recorded in mg/dL and multiplied by ten to give mg/L. Values of zero (32% of measurements) were replaced with the smallest observed positive value. The estimate is sensitive to this choice: substituting a half-detection-limit value moves the adjusted odds ratio from 0.78 to unity, so the index should not be interpreted quantitatively in this dataset |
| CAR | C-reactive protein-to-albumin ratio | CRP (mg/L) / albumin (g/L) | Dimensionless; CRP in mg/L (raw mg/dL × 10) |
| NLR | Neutrophil-to-lymphocyte ratio | Neutrophil / lymphocyte | Dimensionless; both in 10⁹/L |
| SII | Systemic immune-inflammation index | (Platelet × neutrophil) / lymphocyte | In 10⁹/L |
| SIRI | Systemic inflammatory response index | (Neutrophil × monocyte) / lymphocyte | In 10⁹/L |
| PIV | Pan-immune-inflammation value | (Neutrophil × platelet × monocyte) / lymphocyte | In 10⁹/L |
| MHR | Monocyte-to-HDL ratio | Monocyte (10⁹/L) / HDL cholesterol | No unit conversion factor applied |
| TyG | Triglyceride-glucose index | ln[TG (mg/dL) × glucose (mg/dL) / 2] | ln-transformed; dimensionless |
| AIP | Atherogenic index of plasma | log₁₀[TG / HDL] | log₁₀-transformed; dimensionless |
| CHG | Cholesterol–HDL–glucose ratio | (Total cholesterol × glucose) / HDL | All values in mg/dL |
| NHHR | Non-HDL/HDL ratio | (Total cholesterol − HDL) / HDL | Dimensionless |

## Table S3. Component-removal sensitivity analysis across all thirteen indices

Each panel rebuilds the composite outcome after removing the indicated component(s) and refits the adjusted model for every index. Indices are ordered by p-value within each panel.

**Panel A — malnutrition item removed (9 components, high burden ≥ 4; n with high burden = 128)**

| Index | aOR per SD | 95% CI | p | FDR-q |
| --- | --- | --- | --- | --- |
| PNI | 0.638 | 0.476–0.854 | 0.003 | 0.034 ★ |
| HALP | 0.713 | 0.531–0.959 | 0.025 | 0.163 |
| CALLY | 0.764 | 0.573–1.019 | 0.067 | 0.291 |
| NHHR | 1.262 | 0.952–1.674 | 0.106 | 0.344 |
| CHG | 1.233 | 0.914–1.664 | 0.170 | 0.442 |
| AIP | 1.166 | 0.897–1.515 | 0.251 | 0.544 |
| SII | 1.118 | 0.872–1.433 | 0.381 | 0.707 |
| SIRI | 0.897 | 0.665–1.209 | 0.475 | 0.772 |
| MHR | 0.936 | 0.711–1.233 | 0.640 | 0.881 |
| TyG | 1.056 | 0.817–1.365 | 0.678 | 0.881 |
| PIV | 0.968 | 0.748–1.252 | 0.804 | 0.929 |
| NLR | 1.015 | 0.788–1.307 | 0.908 | 0.929 |
| CAR | 0.988 | 0.754–1.295 | 0.929 | 0.929 |

**Panel B — ADL and IADL removed (8 components, high burden ≥ 3)**

| Index | aOR per SD | 95% CI | p | FDR-q |
| --- | --- | --- | --- | --- |
| PNI | 0.779 | 0.596–1.018 | 0.067 | 0.874 |
| HALP | 0.835 | 0.643–1.084 | 0.176 | 0.874 |
| MHR | 0.874 | 0.668–1.143 | 0.326 | 0.874 |
| SII | 1.122 | 0.876–1.436 | 0.362 | 0.874 |
| NLR | 1.098 | 0.859–1.405 | 0.455 | 0.874 |
| CALLY | 0.905 | 0.695–1.180 | 0.461 | 0.874 |
| AIP | 0.911 | 0.708–1.173 | 0.472 | 0.874 |
| NHHR | 0.942 | 0.734–1.210 | 0.639 | 0.874 |
| SIRI | 1.056 | 0.832–1.342 | 0.654 | 0.874 |
| TyG | 0.948 | 0.738–1.216 | 0.673 | 0.874 |
| CAR | 0.955 | 0.719–1.268 | 0.749 | 0.874 |
| CHG | 0.973 | 0.758–1.250 | 0.832 | 0.874 |
| PIV | 1.020 | 0.802–1.296 | 0.874 | 0.874 |

**Panel C — malnutrition, ADL, and IADL removed (7 components, high burden ≥ 3)**

| Index | aOR per SD | 95% CI | p | FDR-q |
| --- | --- | --- | --- | --- |
| PNI | 0.681 | 0.512–0.905 | 0.008 | 0.107 |
| CALLY | 0.742 | 0.554–0.993 | 0.045 | 0.276 |
| HALP | 0.758 | 0.566–1.016 | 0.064 | 0.276 |
| SII | 1.245 | 0.969–1.601 | 0.087 | 0.283 |
| NLR | 1.209 | 0.941–1.552 | 0.138 | 0.359 |
| CHG | 1.177 | 0.903–1.533 | 0.229 | 0.496 |
| SIRI | 1.127 | 0.886–1.434 | 0.331 | 0.615 |
| PIV | 1.105 | 0.869–1.405 | 0.416 | 0.677 |
| NHHR | 1.081 | 0.840–1.392 | 0.546 | 0.788 |
| TyG | 1.060 | 0.820–1.371 | 0.655 | 0.851 |
| MHR | 0.979 | 0.745–1.288 | 0.882 | 0.990 |
| AIP | 0.989 | 0.765–1.279 | 0.935 | 0.990 |
| CAR | 0.998 | 0.765–1.302 | 0.990 | 0.990 |

**Panel D — cognitive item removed (9 components, high burden ≥ 4; n with high burden = 147)**

| Index | aOR per SD | 95% CI | p | FDR-q |
| --- | --- | --- | --- | --- |
| PNI | 0.57 | 0.43–0.77 | <0.001 | 0.003 ★ |
| HALP | 0.64 | 0.48–0.86 | 0.003 | 0.019 ★ |
| CALLY | 0.72 | 0.55–0.96 | 0.024 | 0.103 |
| NLR | 1.27 | 0.97–1.67 | 0.080 | 0.259 |
| SII | 1.24 | 0.95–1.62 | 0.118 | 0.306 |
| CHG | 1.25 | 0.91–1.72 | 0.161 | 0.348 |
| NHHR | 1.15 | 0.88–1.51 | 0.309 | 0.574 |
| SIRI | 1.10 | 0.86–1.40 | 0.463 | 0.749 |
| AIP | 1.09 | 0.84–1.41 | 0.519 | 0.749 |
| PIV | 1.05 | 0.83–1.34 | 0.682 | 0.886 |
| TyG | 1.04 | 0.81–1.34 | 0.769 | 0.904 |
| MHR | 1.02 | 0.78–1.33 | 0.878 | 0.904 |
| CAR | 1.02 | 0.79–1.31 | 0.904 | 0.904 |

No index reached the corrected threshold once both functional measures were removed (Panels B and C), whereas PNI remained significant when only the nutritional component (Panel A) or the cognitive component (Panel D) was dropped. This locates the association in the functional-dependency items. ★ significant after Benjamini–Hochberg correction. aOR, adjusted odds ratio; CI, confidence interval; FDR, false discovery rate; SD, standard deviation. Index abbreviations as in Table S2.

## Table S4. Analysis restricted to patients in whom all ten components were assessed (n = 173)

Because components that were not assessed were counted as absent when the composite was formed, this analysis repeats the primary models in the subgroup with complete ascertainment of all ten components (n = 173; 87 with high burden).

| Index | n | aOR per SD | 95% CI | p | FDR-q |
| --- | --- | --- | --- | --- | --- |
| PNI | 167 | 0.48 | 0.32–0.73 | <0.001 | 0.007 ★ |
| HALP | 167 | 0.52 | 0.35–0.77 | 0.001 | 0.008 ★ |
| SII | 172 | 2.04 | 1.25–3.35 | 0.005 | 0.020 ★ |
| NLR | 172 | 1.96 | 1.20–3.19 | 0.007 | 0.022 ★ |
| PIV | 172 | 1.45 | 0.99–2.14 | 0.059 | 0.154 |
| SIRI | 172 | 1.53 | 0.95–2.44 | 0.079 | 0.171 |
| CHG | 158 | 1.32 | 0.89–1.96 | 0.171 | 0.295 |
| MHR | 162 | 1.27 | 0.89–1.82 | 0.183 | 0.295 |
| CALLY | 149 | 0.79 | 0.55–1.14 | 0.204 | 0.295 |
| NHHR | 160 | 1.10 | 0.77–1.56 | 0.595 | 0.735 |
| AIP | 160 | 1.09 | 0.77–1.55 | 0.622 | 0.735 |
| TyG | 163 | 1.07 | 0.75–1.53 | 0.712 | 0.771 |
| CAR | 150 | 0.97 | 0.67–1.41 | 0.882 | 0.882 |

In this subgroup the PNI and HALP associations were stronger than in the full cohort, and NLR and SII also reached the corrected threshold, which they did not do in the full cohort. Two explanations cannot be separated with these data: counting unassessed components as absent may dilute the burden score and attenuate associations, or patients able to complete every instrument may differ systematically from those who could not. This is addressed in the Discussion. ★ significant after Benjamini–Hochberg correction. aOR, adjusted odds ratio; CI, confidence interval; FDR, false discovery rate; SD, standard deviation. Index abbreviations as in Table S2.

## Table S5. Adjusted odds ratios and prevalence ratios for high burden, all thirteen indices

Prevalence ratios (aPR) were estimated with modified Poisson regression and robust (HC0) variance [S1], because the high outcome prevalence (54.3%) can make odds ratios diverge from prevalence ratios. Indices are ordered as in the main analysis.

| Index | n | aOR per SD (95% CI) | aOR FDR-q | aPR per SD (95% CI) | aPR FDR-q |
| --- | --- | --- | --- | --- | --- |
| PNI | 278 | 0.548 (0.404–0.743) | 0.001 ★ | 0.798 (0.710–0.897) | 0.002 ★ |
| HALP | 278 | 0.635 (0.473–0.852) | 0.016 ★ | 0.817 (0.716–0.933) | 0.018 ★ |
| CALLY | 250 | 0.781 (0.592–1.030) | 0.349 | 0.894 (0.786–1.018) | 0.293 |
| CHG | 261 | 1.316 (0.938–1.845) | 0.365 | 1.065 (1.008–1.125) | 0.107 |
| NLR | 288 | 1.188 (0.911–1.549) | 0.530 | 1.074 (0.980–1.176) | 0.325 |
| SII | 288 | 1.159 (0.889–1.512) | 0.598 | 1.055 (0.976–1.140) | 0.385 |
| NHHR | 264 | 1.134 (0.860–1.497) | 0.692 | 1.040 (0.966–1.120) | 0.551 |
| AIP | 264 | 1.102 (0.847–1.432) | 0.764 | 1.036 (0.943–1.139) | 0.752 |
| TyG | 270 | 1.061 (0.818–1.377) | 0.856 | 1.022 (0.932–1.120) | 0.844 |
| SIRI | 288 | 1.056 (0.828–1.347) | 0.856 | 1.030 (0.926–1.145) | 0.844 |
| MHR | 269 | 1.026 (0.787–1.338) | 0.962 | 1.010 (0.900–1.132) | 0.932 |
| CAR | 251 | 0.987 (0.757–1.287) | 0.962 | 0.995 (0.895–1.106) | 0.932 |
| PIV | 288 | 1.006 (0.789–1.282) | 0.962 | 1.005 (0.908–1.111) | 0.932 |

*The prevalence-ratio analysis agreed with the odds-ratio analysis: only PNI and HALP were significant after correction.*

## Table S6. Incidence rate ratios for the continuous syndrome count (Poisson regression), all thirteen indices

The syndrome count (0–10) was modelled with Poisson regression; the Pearson chi-square/degrees-of-freedom ratio was 1.002, so a negative binomial model was not required. Indices are ordered by p-value.

| Index | n | IRR per SD (95% CI) | FDR-q |
| --- | --- | --- | --- |
| PNI | 278 | 0.888 (0.826–0.954) | 0.012 ★ |
| HALP | 278 | 0.907 (0.854–0.964) | 0.012 ★ |
| CALLY | 250 | 0.922 (0.859–0.989) | 0.097 |
| SII | 288 | 1.049 (1.001–1.099) | 0.153 |
| NHHR | 264 | 1.036 (0.986–1.089) | 0.420 |
| AIP | 264 | 1.038 (0.981–1.097) | 0.427 |
| CHG | 261 | 1.028 (0.982–1.077) | 0.447 |
| NLR | 288 | 1.032 (0.973–1.094) | 0.483 |
| PIV | 288 | 1.013 (0.955–1.075) | 0.926 |
| TyG | 270 | 1.011 (0.954–1.071) | 0.926 |
| SIRI | 288 | 1.004 (0.946–1.065) | 0.926 |
| MHR | 269 | 0.997 (0.940–1.057) | 0.926 |
| CAR | 251 | 0.998 (0.953–1.045) | 0.926 |

## Table S7. Sensitivity of the primary associations to the definition of the comorbidity score

| Comorbidity score definition | PNI aOR (95% CI) | HALP aOR (95% CI) |
| --- | --- | --- |
| Weighted index (primary analysis) | 0.55 (0.40–0.74) | 0.64 (0.47–0.85) |
| Age-adjusted | 0.55 (0.40–0.74) | 0.63 (0.47–0.85) |
| Excluding coronary artery disease | 0.56 (0.41–0.75) | 0.64 (0.47–0.85) |
| Unweighted count of the same conditions | 0.55 (0.41–0.75) | 0.64 (0.47–0.86) |
| Count of all fourteen recorded comorbidities | 0.55 (0.41–0.75) | 0.63 (0.47–0.85) |

*All models were additionally adjusted for age, sex, and polypharmacy, with the index entered as a z-score (per 1-SD). aOR, adjusted odds ratio; CI, confidence interval; HALP, hemoglobin–albumin–lymphocyte–platelet score; PNI, Prognostic Nutritional Index; SD, standard deviation.*

**Figure S1.** Participant flow (STROBE). Of 295 geriatric outpatients with concurrent laboratory testing and comprehensive geriatric assessment, 6 were excluded for missing more than half of the laboratory panel, leaving 289 for analysis. Adjusted index models used complete cases (n = 278).

## Supplementary reference

S1. Zou G. A modified Poisson regression approach to prospective studies with binary data. Am J Epidemiol. (2004) 159:702–6. doi: 10.1093/aje/kwh090
